# The Effect of Three Days of Judo Training Sessions on the Inflammatory Response and Oxidative Stress Markers

**DOI:** 10.2478/v10078-011-0074-1

**Published:** 2011-12-25

**Authors:** Radosław Laskowski, Ewa Ziemann, Robert Antoni Olek, Agnieszka Zembron-Lacny

**Affiliations:** 1 Academy of Physical Education and Sport, Department of Physiology, Gdańsk, Poland; 2 Academy of Physical Education and Sport, Department of Biochemistry, Gdańsk, Poland; 3 Academy of Physical Education Poznan, Faculty of Physical Culture Gorzow Wlkp., Poland

**Keywords:** cytokines, lipid peroxides, muscle damage, sport training

## Abstract

The main purpose of this study was to investigate how extreme physical strain influences cytokine response and oxidative stress markers by examining professional judo athletes during a typical 3-day judo training session (randori combat training).

Creatine kinase (CK) activity, a marker of muscle damage, was considerably elevated immediately after randori training. Pro- (IL-1β and TNF-α) and anti-inflammatory (IL-6 and IL-10) cytokines were also increased. The strongest effect was seen in IL-1β concentration, which correlated with CK activity (r = 0.49, P < 0.05). All the observed cytokines returned to baseline (IL-1β) or even dropped below initial levels (TNF-α, IL-6 and IL-10) 12 h after completing the training. Lipid peroxides (LPO), a marker of reactive oxygen species, also decreased below their initial values. LPO levels correlated directly with IL-1β, TNF-α, IL-6 and IL-10.

This study is the first to evaluate the effect of a 3-day judo training session on muscle damage by evaluating the release of pro- and anti-inflammatory cytokines and markers of oxidative stress. It is also the first to demonstrate significant changes in the blood cytokine profile that correlate with lipid peroxide levels and muscle damage.

## Introduction

Frequent injuries within groups of elite athletes may indicate that thorough recovery has not been possible. In a wide range of athletic disciplines, long competitive seasons are followed by short recovery during the post season. After only three to four weeks of recuperation, endurance training prior to the start of the following season begins. This mode of training is associated with significant systemic hormonal and inflammatory effects. Evidence for this was presented by Reinke et al. (2009), who suggested that the immune system is not able to function efficiently during periods of high physical stress. Main et al. (2010) demonstrated that eight weeks of intense endurance training in rowers induced an overtraining syndrome that was associated with changes in IL-1β, IL-6 and TNF-α levels. Mochida et al. (2007) postulated that an overtraining state might be predicted by measuring neutrophil function, including reactive oxygen species (ROS) production, phagocytic activity and serum opsonic activity.

The number of neutrophils increases with exercise. The degree of the increase in neutrophil count depends mostly on the intensity of activity (Fry et al., 1992). Because neutrophils can generate ROS, this elevation may represent an anti-inflammatory reaction (Pyne, 1994). ROS can have both beneficial and detrimental effects. They destroy invading microorganisms but can also cause oxidative damage to normal body tissues and organs. For example, ROS can cause degeneration of muscle tissues that were injured during exercise. Umeda et al. (2008) reported that, after a typical 2 h judo training session, the levels of IgG and IgA significantly increase. Increases in IgG and IgA, as well as increases in serum enzymes and neutrophils, suggest that exercise-mediated muscle degradation may trigger the anti-inflammatory response. In the same study, the authors reported that neutrophils stimulate ROS production during exercise. This fact, along with the increased neutrophil count, suggests that a 2 h judo training session induces changes in the markers of oxidative stress.

Therefore, we hypothesised that the physical demands imposed upon athletes during an intensive training session may influence immune function to such an extent that the recuperation period is insufficient to allow a reduction in stress-induced tissue damage. The aim of this study was to investigate how extreme physical strain influences inflammatory mediators by examining professional judo athletes during a 3-day training period.

## Material and Methods

### Subjects

Eleven members of the national judo team participated in the study. Participants had an average of 11.0 ± 2.0 years of judo training and had a training workload of 10 sessions per week during the period of this study. All judokas lived in the same accommodations. Athletes were not taking any antioxidant supplements (vitamins or minerals) at the time of the study. Diets did not include more than 4200 calories per day, with protein intake varying from 1.3 to 1.5 g per kg of body mass.

All the subjects were informed of the aims of the study and gave written consent for participation in the project. The protocol of the study was approved by the ethics committee at the Medical University Poznan, in accordance with the Helsinki Declaration. Table 1 presents the schedule of investigations performed in this study.

### Anthropometric measurements

Body mass (BM) and body composition were estimated using a bioelectrical impedance floor scale (TBF-300 Body Fat Monitor/Scale Analyzer, Tanita, Japan), calibrated in accordance with the manufacturer’s guidelines, prior to each test session. One hour after a light breakfast, participants voided their bladder and bowels. Following this, duplicate measurements were taken with participants standing and wearing only briefs, as recommended by the guidelines. The average of these two measurements was used for the final analysis (Table 2).

Additionally, two weeks before the trial, aerobic and anaerobic capacities were determined on separate days. The tests were performed on a mechanically braked cycle ergometer (884E Sprint Bike, Monark, Sweden) as follows. Aerobic capacity was determined during the VO_2_max test. Immediately following a warm-up period (5-min at 1.5 W · kg^−1^), participants began cycling at workloads that gradually increased by 25 W · min^−1^ until volitional exhaustion. Breath-by-breath pulmonary gas exchange was measured (Oxycon-Pro, Jaeger-Viasys Health Care, Germany) throughout the test (Ziemann et al., 2010). The anaerobic capacity protocol started with a standard 5-minute warm-up at a workload of 1.0 W·kg^−1^. After the warm-up, all subjects performed a 30 s all-out supramaximal test. Flywheel resistance equalled 0.075 kG per kg of body mass (corresponding to 7.5% of an individual’s body weight). Subjects initiated the test from a dead stop with the resistance level on the ergometer’s friction belt preset by the laboratory staff immediately before the Wingate test (Bar Or, 1987; Olek et al., 2010).

### Blood collection

Blood samples were taken from the antecubital vein at rest, immediately after training and 12 h after the training session (Table 1). Samples were collected in anticoagulant-containing (EDTAK_2_) single-use containers. After collection, the samples were immediately cooled to 4° C. Within 10 min, samples were centrifuged at 3000 g at 4° C for 10 min. Aliquots of plasma were stored at −80° C.

### Muscle damage

Plasma creatine kinase (CK) activity was used as the marker of muscle damage and was evaluated by standard kit (Emapol, Gdańsk, Poland). The CK detection limit for this kit was 6 U · l^−1^. The intra-assay CV for the CK kit was 1.85%.

### Pro- and anti-inflammatory cytokines

Plasma interleukin (IL-1β, IL-6, IL-10) and (TNF-α) levels were determined by enzyme immunoassay methods using commercial kits (R&D Systems, USA). Detection limits for IL-1β, TNF-α, IL-6 and IL-10 were 0.023, 0.038, 0.039 and 0.500 pg · ml^−1^, respectively. The average intra-assay CV was < 8.0% for all cytokines.

### Reactive oxygen species (ROS)

Levels of plasma hydrogen peroxide (H_2_O_2_) and lipid peroxide (LPO), which are markers of ROS activity, were determined with the Oxis Research kits (USA). H_2_O_2_ and LPO detection limits were 6.25 nmol · ml^−1^ and 0.1 nmol · ml^−1^, respectively. The intra-assay coefficients of variation (CV) for the H_2_O_2_ kit and for the LPO kit were both < 10%.

### Statistical analysis

Statistical calculations were performed using STATISTICA 9.0. Statistical significance was assessed by repeated analysis of variance (ANOVA) and Tukey’s post-hoc test. Associations among measured parameters were analysed using Pearson’s linear regression analysis. Statistical significance was set at *P* < 0.05. Results are expressed as a mean and standard deviation (x ± SD).

## Results

### Muscle damage

CK activity, which is a marker of muscle damage, was considerably increased directly after randori training. CK levels remained elevated compared to baseline 12 h after the last training session (Table 3).

### Anti- and pro-inflammatory cytokines

**Figure 1:**
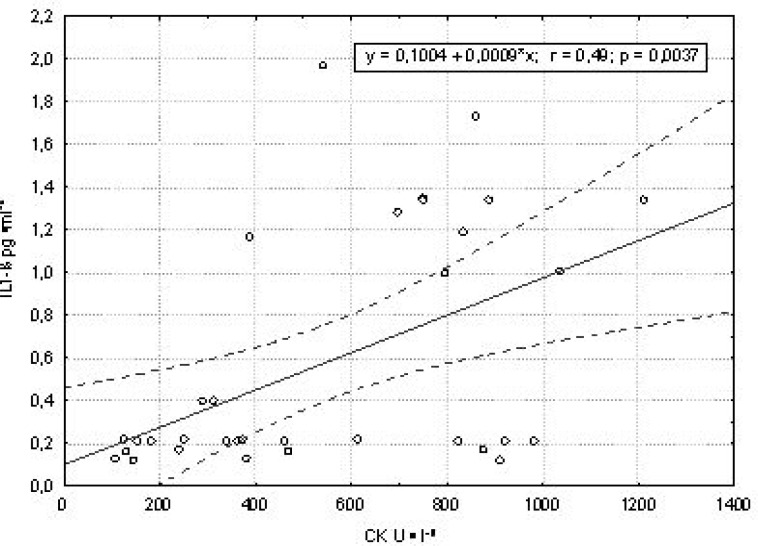
The relationships between the concentration of the IL-1β and CK activity

Changes in the levels of pro- and anti-inflammatory cytokines were also observed (Table 3). The strongest effect was seen in IL-1β concentration, which correlated with the CK activity (r = 0.49, *P* < 0.05; Figure 1). Immediately after randori training, the pro-inflammatory cytokines IL-1β and TNF-α increased 6.5 and 1.1-fold. IL-6 and IL-10 increased 3.8 and 1.4-fold respectively (Table 3), and their levels were highly correlated (r = 0.669, *P* < 0.05; Table 4). Twelve hours after training, IL-1β returned to baseline, whereas all other cytokine levels dropped below their initial values (Table 3).

### Reactive oxygen species

H_2_O_2_ concentration tended to increase immediately after randori training and at 12 h after training, but the difference was not statistically significant. LPO concentrations did not change immediately after training and descended below their initial values 12 hours after completing training (Table 3). LPO levels directly correlated with levels of all the cytokines analysed (Table 4).

## Discussion

Qualification rounds in judo for the Olympic Games involve a number of tournaments that occur with a very high frequency at particular times of the year. Consequently, professional judo fighters are forced to undergo a very intensive competitive season and participate in a wide range of events. Such a high intensity of competition may lead to overtraining, observable muscle damage and an increased inflammatory response. Therefore, the training period must prepare professional athletes for adapting to changing conditions during an intense competitive season.

We observed that the 3-day training session commonly used in judo practice (including randori combat training) caused muscle damage. CK activity increased over baseline immediately after the last training session and CK levels remained significantly elevated after 12 hours of rest. These levels are higher than those observed by Umeda et al. (2008), who demonstrated a non-significant increase in CK to 293.9 ± 112.3 IU · l^−1^ after standard judo training consisting of a warm-up, 70 min of randori and a cool-down (Umeda et al., 2008). The much higher values observed in our study may result from the accumulation of muscle damage induced by consecutive training sessions.

The amount of pro- and anti-inflammatory cytokines released after a single physical effort depends on the intensity and time of the activity performed, as well as the muscle groups involved in the exercise. The most dramatic variations have been recorded in a response for endurance-focused activities lasting longer than 2 hours, since the level of IL-6 increased 120-fold, IL-10 60-fold, TNF-α 3-fold and IL-1β 3-fold. On the other hand, submaximal concentric exercise lasting up to 2 hours resulted in less significant alterations, with the level of, IL-6 increasing 5-fold and TNF-α by 50%. No change in IL-1β was noted in these conditions. Finally, concentric physical efforts lasting up to 30 minutes as well as short eccentric activities induced an observably weaker cytokine response, since the level of IL-6 increased 2-fold maximally. Increases in IL-10 by 25%, IL-1β by 17% and a constant or decreasing value of TNF-α were noted (Hirose et al., 2004; Ostrowski et al., 2004; Peake et al., 2005; Suzuki et al., 2002). The presence of pro-inflammatory cytokines, such as TNF-α and IL-1β, is necessary to stimulate the production of IL-6 and growth factors that initiate satellite cell proliferation and reconstruction of muscle fibres (Collins and Grounds, 2001; Reid and Li, 2001).

Levels of pro- and anti-inflammatory cytokines increased immediately after the last training session (Table 3). However, relatively small alterations were noted in the concentrations of TNF-α and IL-1β (with 6.5-fold and 1.1-fold increases, respectively). The concentrations of both these cytokines dropped 12 hours after randori training, IL-1β level returned to baseline and TNF-α level dropped below their initial levels. Simultaneously, as a response to muscle damage and its associated pro-inflammatory reaction, synthesis of the anti-inflammatory cytokines IL-6 and IL-10 was observed. The rapid response observed among participants in this study indicates their well-developed ability to adapt to the very high eccentric and concentric muscle contractions that characterise judo training. According to Parmigniani et al. (2006), the level of IL-6 significantly increased after randori training (P<0.05), but IL-1β remains unaffected by competition.

LPO and H_2_O_2_ are the most widely used markers of oxidative stress. Plasma LPO level is an empirical measurement of the complex process of peroxidation after a single period of exercise or athletic training. In our study, randori training did not appear to induce an elevation in H_2_O_2_ nor in LPO (Table 3). Previous studies have demonstrated that physical training prevents exhaustive exercise-induced oxidative stress by upregulating the antioxidant system (Sentürk et al. 2001; Oztasan et al. 2004). Moreover, Finaud et al. (2006) showed that competition induced similar changes in the oxidant-antioxidant status regardless of dietary intake during the seven days before a competition. The effect of competition on antioxidant and oxidant parameters therefore appears to be more pronounced than that of diet.

A recent study by Zembron-Lacny et al. (2010) indicated that the combination of prolonged exercise with an eccentric workload enhances ROS production as well as the levels of muscle-derived cytokines. A strong correlation between anti-inflammatory cytokines (IL-6, IL-10), hydrogen peroxide and 8-isoprostanes was also reported in that study. We did not observe a correlation between hydrogen peroxide and cytokine levels. However, our data show that LPO levels are correlated with both anti- and pro-inflammatory cytokine levels (Table 4). These observed differences may be the result of the nature of judo practice. Judo is characterised by high-intensity, short-duration exercise and it requires the athlete to use a combination of aerobic and anaerobic capacities (Franchini et al., 2009). Thus, different types of metabolism and energy substrates are utilized in judo (Degoutte et al., 2003). Some studies have shown significantly increased ROS levels after judo training (Miura et al., 2005). However, some researchers have observed that ROS compensate for each other following acute exercise, with an inverse ratio change (Sugawara et al., 1999).

In the preparatory phase of training (or the pre-season), anaerobic and aerobic capacities both play an important role. The average recorded maximal oxygen uptake has been reported to vary among athletes of different nationalities. In high-level US judo athletes, the recorded maximal oxygen uptake was 53.2– 55. 6 ml kg^−1^ min^−1^ (Callister et al., 1990; Callister et al., 1991), 49.9 ml kg^−1^min^−1^ in Japanese judo athletes (Ebine et al., 1991), 57.6 ml kg^−1^ min^−1^ in Canadians (Little, 1991); 59.8 ml kg^−1^ min^−1^ in French judo athletes (Majean and Gaillat, 1986). According to Franchini et al. (2011), typical maximal oxygen uptake values oscillate around 50–55 (ml kg min^−1^) for male judo athletes. The values recorded in the present study were similar, reaching an average of 54.0 ml kg^−1^ min^−1^ (Table 2). Sbriccoli et al (2007) noted a peak power level of 12.1 W kg^−1^, Sterkowicz et al. (1999) observed peak power of 11.4 W kg^−1^ and French judo athletes were characterized by 14.6 W kg^−1^ (Gariod et al., 1995). The maximal anaerobic power recorded in our participants is similar to previously reported values (Table 2). However, we did not repeat these measurements, because we did not expect any change after the short period of training in this study.

Our study is the first to evaluate the effect of a 3-day judo training session on muscle damage by measuring levels of pro- and anti-inflammatory cytokines and markers of oxidative stress. We demonstrate that judo training induces significant shifts in the blood cytokine profile. These changes in blood cytokine levels correlate with the lipid peroxide levels. This report also confirms cytokines as effective markers of normal recovery after intense exercise.

## Figures and Tables

**Table 1:** Training program during the study

	**a.m.** 11:00 – 12:00	**Intensity** (% HR max)	**p.m.** 17:30 – 19:00	**Intensity** (% HR max)

**Monday**	Rest *blood collection*		Training A	50 – 60
**Tuesday**	Training B	100 – 120	Training A	70 – 80
**Wednesday**	Training C	50 – 60	Training D *blood collection*	70 – 100
**Thursday**	Rest *blood collection*		Training A	40 – 50

Rest: Take a rest or attend the lectures;

Training A: Judo training for practice (technique and tactics);

Training B: Interval training consisted of sprint running (4× 50 m, 4× 100m, 2 × 200m and jogging, rest between each repetition was 1min, 3min, 3min appropriately and 10 min rest between series);

Training C: Distance running for 30 minutes;

Training D: Judo training for practice (randori – combat training 5min x 10, rest between series 2 min)

**Table 2:** Anthropometric and physical (aerobic and anaerobic parameters) characteristics of judoists

Variable	Values are means ± SD
Age (yr)	22.1 ± 2.5
Body height (cm)	182.0 ± 4.5
Body mass (kg)	83.7 ± 8.3
FFM (kg)	75.2 ± 5.6
Fat (kg)	8.5 ± 3.7
Fat %	9.9 ± 3.2
BMI (kg/m^2^)	25.2 ± 1.5
VO_2_max (l· min^−1^)	4.4 ± 0.4
VO_2_max (ml · kg^−1^·min^−1^)	54.0 ± 4.0
Work output (J · kg^−1^)	263.7 ± 7.6
Max Power (W · kg^−1^)	11.9 ± 0.8

Fat = fat mass(%, kg), FFM = free fat mass, BMI = body mass index, VO_2_max - maximal oxygen uptake in absolute and relative values, Work output and Max Power measured during Anaerobic Wingate Test

**Table 3:** *Levels of plasma hydrogen peroxide (H_2_O_2_), lipid peroxides (LPO), interleukin-1β (IL-1β), tumour necrosis factor α (TNFα), interleukin-6 (IL-6), interleukin-10 (IL-10), and creatine kinase (CK)*

**Variable**	**Rest**	**Directly after last training session**	**Twelve hours after last training session**
CK IU · l^−1^	211 ± 91	795 ± 223*	648 ± 258 *
IL-1β pg · ml^−1^	0.2 ± 0.0	1.3 ± 0.3 *	0.21 ± 0.07 #
TNFα pg · ml^−1^	3.8 ± 0.5	4.2 ± 0.2	1.01 ± 0.22 *#
IL 6 pg · ml^−1^	1.2 ± 0.5	4.6 ± 1.3 *	0.62 ± 0.19 *#
IL 10 pg · ml^−1^	13.2 ± 3.1	18.3 ± 3.3 *	9.38 ± 1.33 *#
H_2_O_2_ μmol · ml^−1^	8.9 ± 3.0	11.4 ± 2.9	10.33 ± 1.80
LPO nmol · ml^−1^	3.4 ± 0.8	3.3 ± 0.5	1.7 ± 0.2 *#

Values are means ± SD, significant differences (P<0.05)

* vs. rest ;

# vs. directly after randori training

**Table 4:** *Correlations between hydrogen peroxide (H_2_O_2_), lipid peroxides (LPO) and cytokines*

	IL-1β pg · ml^−1^	TNFα pg · ml^−1^	IL- 6 pg · ml^−1^	IL-10 pg · ml^−1^
H_2_O_2_ μmol · ml^−1^	0.28	0.09	0.13	0.15
LPO nmol · ml^−1^	0.42*	0.84*	0.47*	0.51*

* presented correlations are significant statistically (P<0.05)
